# Current state of the art and recommendations in robotic mitral valve surgery

**DOI:** 10.1093/icvts/ivac160

**Published:** 2022-06-24

**Authors:** Meindert Palmen, Emiliano Navarra, Johannes Bonatti, Ulrich Franke, Stepan Cerny, Francesco Musumeci, Paul Modi, Sandeep Singh, Elena Sandoval, Matteo Pettinari, Patrique Segers, Monica Gianoli, Frank van Praet, Herbert de Praetere, Jan Vojacek, Theodor Cebotaru, Burak Onan, Cengiz Bolcal, Cem Alhan, Ahmed Ouda, Ludovic Melly, Ghislain Malapert, Louis Labrousse, Alfonso Agnino, Tine Phillipsen, Jean-Luc Jansens, Thierry Folliguet, Piotr Suwalski, Koen Cathenis, Fabien Doguet, Anton Tomšič, Wouter Oosterlinck, Daniel Pereda

**Affiliations:** Leiden University Medical Centre, Leiden, Netherlands; Cliniques Universitaires Saint Luc, Brussels, Belgium; University of Pittsburgh Medical Center (UPMC), Pittsburgh, PA, United States; Robert Bosch Hospital, Stuttgart, Germany; Na Homolce Hospital, Prague, Czech Republic; San Camillo Hospital, Rome, Italy; Liverpool Heart and Chest, Liverpool, United Kingdom; ISALA Hospital, Zwolle, Netherlands; Hospital Clínic de Barcelona, Barcelona, Spain; Ziekenhuis Oost Limburg, Genk, Belgium; Maastricht University Medical Center, Maastricht, Netherlands; University Medical Centre Utrecht, Utrecht, Netherlands; OLV Ziekenhuis, Aalst, Belgium; Imelda Hospital Bonheiden, Bonheiden, Belgium; University Hospital Hradec Kralove, Hradec Kralove, Czech Republic; MONZA Hospital, Bucharest, Romania; Istanbul Mehmet Akif Ersoy Cardiovascular Surgery Hospital, University of Health Sciences, Istanbul, Turkey; Gulhane Education ve Research Hospital, Ankara, Turkey; Acibadem Maslak Hospital, Acibadem University, Istanbul, Turkey; University Hospital Zurich, Zurich, Switzerland; CHU UCL Namur, UC Louvain, Godinne, Belgium; CHU Dijon, Dijon, France; University Hospital Bordeaux, Bordeaux, France; Humanita Gavazzeni, Bergamo, Italy; University Hospital Ghent, Ghent, Belgium; Erasme Hospital Brussels, Brussels, Belgium; Henri MONDOR Hospital, Assistance Publique Hopitaux de Paris, Creteil, France; Central Clinical Hospital of the Ministry of the Interior and Administration, Centre of Postgraduate Medical Education, Warsaw, Poland; AZ Maria Middelares, Ghent, Belgium; Cardiovascular Institute Paris Sud, Private Hospital Jacques Cartier, Massy, France; Leiden University Medical Centre, Leiden, Netherlands; Department of Cardiovascular Sciences, University Hospital Leuven, KU Leuven, Leuven, Belgium; Hospital Clínic de Barcelona, Barcelona, Spain

##  

The adoption of robotics in cardiac surgery is increasing rapidly [1]. The unparalleled range of motion and precision combined with 3-dimensional (3D) vision in a minimally invasive surgical setting facilitates complex cardiosurgical procedures without compromising on the surgical techniques utilized. This unique combination of innovative characteristics holds the promise to improve outcome in various groups of patients. Until the recent surge in robotic cases, the penetrance of robotics in cardiac surgery has been low for decades, likely related to the high initial costs in combination with a lack of structured training and mentoring programs. Furthermore, the new European Medical Device Regulation may hamper further adoption of this technique because it seems to lead to the paradoxical withdrawal of robotic tools from the market rather than the anticipated improvement in quality.

Scientific data on the long-term outcomes of robotic cardiac surgery are scarce compared to the data from conventional surgical approaches. The recent initiation of a European registry of robotic cardiosurgical procedures and several European research initiatives addressing these issues will be the next important step towards the international benchmarking of this promising surgical technique and, more importantly, towards recognition of robotic cardiac surgery as a valuable additional treatment modality.

To address these issues and to conquer the challenges ahead, European robotic cardiac surgery centres have recently combined forces by initiating a European Association for Cardio-Thoracic Surgery-endorsed European Robotic Cardiac Surgery Task Force.

Robotics entered the field of surgery more than 2 decades ago. Although the first surgical robots were originally developed to enable surgeons to treat victims of war at a safe distance from the battlefield, they proved to be most suitable for performing complex surgical procedures in a minimally invasive on-site surgical setting. In the past 2 decades, after the pioneering work of brave and brilliant surgeons, robotics have been gradually adopted in cardiothoracic surgery and earned their role in cardiosurgical practice. After a slow initial adoption during the first decade or so, the variety of cardiac procedures and the number of cases have been steadily increasing over the past decade, as has the scientific attention paid to these procedures. Nowadays, robotic instruments are used mainly in minimally invasive mitral valve surgery and coronary revascularization (mostly robotic-assisted minimally invasive direct coronary artery bypass and totally endoscopic coronary artery bypass at a few specialized centres), although the surgical palette is still expanding.

Several large studies have demonstrated robotic mitral surgery to be a safe, feasible, durable and highly reproducible technique, with reduced perioperative blood loss, less surgical trauma and shorter lengths of stay, enabling swift patient recovery and return to everyday life [1, 2]. Despite these encouraging results, adoption of robotics in surgical practice is still hampered by practical issues. Initial investment in hardware and the additional procedural costs may be formidable obstacles, although reduced hospital length of stay and less frequent administration of blood products could compensate for this. Furthermore, overcoming the initial learning curve in mastering the robotic instruments in a new surgical environment necessitates a structured and safe teaching and proctoring program, with the recommended use of the dual console throughout the training period, that should preferably be developed independent of industry [3]. Acceptance of robotic mitral valve surgery as a distinct but complementary treatment option for selected patients may be aided by the set-up of a European database, facilitating the benchmarking of individual robotic centres and improving quality control and procedural safety for the individual patient.

##  

Surgical mitral valve repair is the gold standard for the treatment of severe primary mitral insufficiency. Based on Carpentier’s principles of the “French correction”, initially described almost 4 decades ago [4], surgical techniques have been perfected ever since, resulting in excellent clinical outcomes and repair durability, restoring patients’ life expectancy and quality of life [5, 6]. Simultaneously, the advent of minimally invasive mitral valve surgery using video-assisted port-access techniques in conjunction with peripheral extracorporeal circulation managed to reduce surgical trauma, thereby enabling swift patient recovery and reducing the duration of hospital admissions.

The theoretical disadvantages of port-access video-assisted mitral valve repair include the lack of 3D vision and the restrictions in dexterity of surgical instruments in this setting. Robotic and port-access mitral valve surgery are based on comparable concepts: Both offer patients the benefits of minimally invasive surgery. Robotic surgery relies on sophisticated technological solutions to allow any type of repair technique to be performed. High-quality comparative analyses of early and, more importantly, late patient- and valve-related outcomes are needed to establish the efficacy of both techniques. An important advantage of robotic surgery that is likely to play an important role in the future is the fact that robotic platforms offer possibilities for training purposes. Dual consoles allow direct supervision and facilitate high-quality training of residents and young surgeons under the supervision of an experienced surgeon.

The discussion has shifted from the feasibility of minimally invasive mitral surgery towards further optimalization of the technical and safety aspects of valve repair. In some patients with unfavourable anatomies, these technical improvements are decisive in determining the feasibility of a durable repair in a minimally invasive setting. Meanwhile, intermediate and long-term results of robotic mitral valve repair have been described that demonstrate an at least equal efficacy and durability compared with their non-robotic minimally invasive counterparts [1, 2, 7]. Although the debate on the superiority of either robotic or port-access mitral valve surgery is ongoing and surgeons passionately defend their approach of choice, it would be a wiser approach to value both techniques as distinct but complementary treatment options for selected patients. Both approaches were designed to offer patients a safe and effective state-of-the-art type of mitral valve surgery while reducing surgical trauma and improving patient recovery.

##  

Robotic mitral surgery has been proven to be an effective and safe technique and, with adequate experience, may be used for all indications and subsets of patients, regardless of the complexity of the mitral lesions. This situation is in great part due to the fact that all the techniques used in conventional surgery can be reproduced with the robotic system with great fidelity, assuring that the quality of the intervention is not compromised in any way.

##  

Robotic mitral surgery follows the same general indications as conventional surgery, as described in the current European Association for Cardio-Thoracic Surgery/European Society of Cardiology guidelines for the management of valvular heart disease [8]. As with conventional surgery, all patients evaluated for robotic mitral surgery should undergo a comprehensive preoperative assessment, including a clinical history, physical examination, 12-lead electrocardiogram and appropriate imaging studies, including high-quality transthoracic and preferably also transoesophageal echocardiography. Additionally, all patients should receive electrocardiogram-gated volumetric computed tomography angiography of the chest, abdomen and pelvis before surgery for the assessment of the thoracic and abdominal aorta to rule out significant atherosclerotic disease that may result in serious perioperative vascular complications. The use of a patient-driven algorithm, as proposed by Gillinov *et al.* [2], could provide an effective tool to avoid all predictable complications.

Robotic mitral surgery has some general contraindications that should be actively sought for during the preoperative evaluation:

- Coronary artery disease requiring surgical revascularization,
- Severe peripheral vascular disease or aneurysms of the descending thoracic or abdominal aorta,
- Prior right chest surgery, radiation or trauma,
- Unusually small thoracic cavity,
- Severe chest wall deformities, such as scoliosis and pectus excavatum,
- Ascending aorta dilatation >45 mm or calcification,
- Moderate to severe aortic stenosis or regurgitation,
- Severe calcification of the mitral valve annulus, and
- Severe pulmonary dysfunction or pulmonary hypertension.

Most of these items are relative contraindications, and, in selected patients, procedural modifications can be introduced to facilitate a robotic approach. As an example, robotic mitral surgery is facilitated by single-lung ventilation at different moments during the operation. Patients with severe chronic pulmonary disease or pulmonary hypertension should be evaluated carefully to determine if they can tolerate this safely. In some patients, single-lung ventilation can be reduced or even avoided at the expense of longer duration of cardiopulmonary bypass (CPB) [9].

##  

Conventional general anaesthesia with endotracheal intubation can be performed either with a double-lumen endotracheal tube or with a single lumen tube and a bronchial occluder. The patient is placed in a supine position and the right chest is slightly elevated. The right arm can be positioned on the right side below the right chest or abducted laterally to expose the lateral chest wall. In either case, care must be taken to avoid neural injuries due to compression.

CPB is commonly instituted with cannulation of the right common femoral artery and vein, using a small incision to expose both vessels. The Seldinger technique and transoesophageal guidance are encouraged to minimize vascular complications during cannulation. In cases when an arterial cannula that completely occludes the lumen is needed (as in obese patients with poor femoral arteries) and a long procedure time is anticipated, a distal perfusion line is placed to prevent limb ischaemia and postoperative compartment syndrome. For this purpose, a 6 Fr introducer connected to the arterial line through a side port can be used.

Routine cannulation of the jugular vein is not mandatory for isolated mitral surgery. For most robotic cases it is only indicated for (concomitant) tricuspid valve surgery or for very tall patients in whom the superior caval vein cannot be adequately drained by the commercially available long venous cannulas. The use of controlled vacuum-assisted systems may optimize venous drainage and visualization. Continuous CO_2_ insufflation inside the right chest is extremely useful to prevent air embolization and reduces camera fogging. Care should be to avoid over-pressurizing the thoracic cavity, especially in the case of small ports.

##  

**Figure 1:**
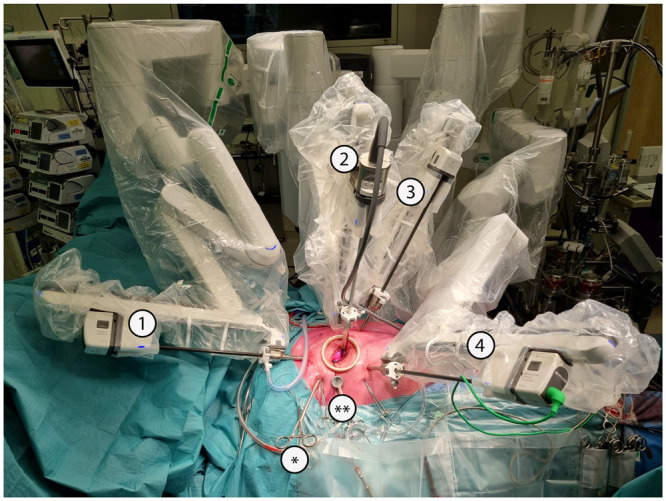
Robotic system (Intuitive Da Vinci X) docked to the patient. It is important to connect all 4 arms in the direction they will move, to avoid conflict with and between other structures, both inside and outside the chest. The camera port is connected to arm number 2 (but can also be connected to any of the other arms in the newer systems) and, under endoscopic or direct visualization, surgical instruments operated by the right and left hands of the console surgeon are typically placed in arms number 1 and 4 and introduced by the patient side assistant for control by the console surgeon, depending on the system used. The atrial retractor is inserted under endoscopic vision on the remaining arm (arm number 3). * The transthoracic aortic clamp is inserted through a separate stab wound incision. ** A suction catheter to vent the left heart chambers can be inserted through the working port or via a separate port.

Different port configurations and sequences of steps can be used with small differences between them, depending on the characteristics of the patient, the robotic system used and the preferences of the surgical team (Fig. 1). Basically, under single left-lung ventilation, the working port is created in the third or fourth intercostal space by performing a 1.5- to 4-cm long skin incision. A soft-tissue retractor is placed to prevent fatty tissue or debris from entering the heart during the procedure. Then, an 8-mm trocar is placed more anteriorly in the same intercostal space to serve as the camera port. Alternatively, the 3D-camera can be introduced through the anterior edge of the working port. Under thoracoscopic visualization, the right arm trocar is inserted 2 intercostal spaces caudally to the working port and the left arm trocar, 1 intercostal space cranially. Finally, the trocar for the left atrial retractor is inserted in the fifth or sixth intercostal space just medial to the mid clavicular line, avoiding injury to the internal thoracic artery. The CO_2_ infusion line can be connected to any of the ports and switched between them as best determined for each port configuration. A suction catheter to vent the left heart chambers and the cardioplegia line can be inserted through the working port or via a separate incision, as preferred. Depending on the patient’s anatomy, a diaphragm retraction suture may be required. This suture, together with the pericardial traction sutures, is pulled and exteriorized below the level of the working port. Once all thoracic steps are completed, the femoral vessels are cannulated and connected to the CPB circuit, and the robotic system can be docked to the patient.

##  

Transthoracic aortic cross-clamping and endoaortic balloon occlusion are the 2 main strategies used for aortic cross-clamping and myocardial protection. Both have proven their efficacy with excellent results, and the choice for 1 or the other technique is based on surgeon and team preference and habit. The endoaortic balloon avoids using the additional incision required for the external clamp and aortic manipulation for root cannula insertion and provides a less crowded surgical field. However, it requires specific training and the participation of several team members (surgeon, scrub nurse, perfusionist and anaesthesiologist), careful monitoring throughout the entire procedure and a refined technique that comes with its own learning curve. In addition, in patients with a high body mass index, due to the encumbrance on the endoballoon inside the femoral cannula, a second peripheral arterial cannulation can be required to secure adequate peripheral perfusion with adequate flow and perfusion pressures. In contrast, the transthoracic clamp is simpler, does not require continuous monitoring and reduces the costs of the intervention, but does require another stab incision in the chest and may create internal conflicts. However, this can be avoided by using flexible (Cygnet) or detachable (Glauber) clamps, introduced through the working port itself. Cardioplegic arrest can be obtained with antegrade blood or crystalloid cardioplegia, according to the preferences of the surgical team, with or without concomitant administration of retrograde cardioplegia.

##  

Access to the mitral valve is established through a left atriotomy with an incision in Waterston’s (Sondergaard's) groove. The heart is approached from the lateral position with direct view of the mitral valve; therefore, excessive heart manipulation is not needed. The left atrial retractor is used to facilitate optimal valve exposure and can easily be repositioned at any time during the operation, as needed. As in open surgery, annular sutures placed in the posterior part of the mitral valve annulus can be used to further optimize valve exposure. The combination of available tools and high-quality 3D vision provides an excellent view of the mitral valve at all times during the operation.

##  

Robotic mitral surgery has important differences compared to conventional surgery or other minimally invasive approaches that involve changes in the techniques, skills and tools used. These include the console for the remote control of the robotic system, 3D camera visualization of the surgical field and specifically designed surgical instruments. Furthermore, the use of the dynamic (left atrial) retractor, specifically designed for robotic mitral surgery, is an important tool that allows excellent exposure of the entire mitral valve while minimizing the geometric distortion and manipulation of the leaflets.

Mitral surgery should follow the exact same basic principles regardless of the approach used in order to ensure a high repair rate and good long-term results. Most of the techniques used in conventional surgery can be used successfully in robotic surgery, but some adaptations must be implemented to facilitate their use in robotic mitral repair. The ultimate objective should be to reproduce the techniques used in conventional surgery and achieve at least the same quality of results.

**Triangular and quadrangular leaflet resections.** Triangular resections are well-suited for robotic surgery. The robotic platform provides both excellent visualization and instrument dexterity to perform precise leaflet resections. Reconstruction of the leaflet can be accomplished with interrupted or running sutures with all types of suture material.

Quadrangular resections are preferred when large portions of the posterior leaflet are prolapsing with a great excess of tissue. In complex cases, the “sliding plasty” technique allows reconstruction without the need for excessive annular plication. This procedure can be accomplished with the robot with extreme accuracy.

**Chordal replacement.** Neochordal implants using polytetrafluoroethylene sutures has become extremely popular in the last 2 decades due to its simplicity, flexibility, reversibility and good long-term durability. Neochords can be constructed and implanted with the robot reproducing all the technical variations currently in use, including the use of preformed loops. The robotic platform offers excellent visualization of the subvalvular apparatus by lifting up the anterior mitral valve leaflet using the left atrial retractor, and the wristed instruments allow for the precise placement of the sutures through the papillary muscle and leaflet.

**Mitral annuloplasty.** All types of annuloplasty bands and rings can be implanted robotically as indicated by the characteristics of the patient and the preference of the surgeon. Sizing of the ring follows the same standardized method as that used in conventional surgery. The implantation can be performed using interrupted or running sutures. Titanium fasteners (Cor-Knot device, LSI Solutions, Viktor, NY, USA) have enabled fast anchoring of the ring or band.

Implantation of an annuloplasty band can be performed by a continuous suture. In case a ring needs to be implanted, the ring can be held outside or inside the thoracic cavity during the implant process. When the ring is held within the thoracic cavity, the annuloplasty ring is first positioned in the left atrium. One after another, the annuloplasty sutures are first passed through the mitral valve annulus and then directly through the ring. The sutures are tied using titanium fasteners before the next suture is placed. When the ring is held outside the thoracic cavity, the implant process resembles that of a standard implant. This process usually results in a slightly larger working port incision.

**Mitral valve replacement.** As with annuloplasty devices, all types of mitral prostheses can be implanted robotically, both mechanical and tissue valves. The robot allows all types of replacement techniques, including partial or complete chordal sparing and intra- or supra-annular positioning. Annular decalcification and debridement can also be accomplished in cases of mitral annular calcification or endocarditis (specific resection tools are still to be developed to deal with heavy calcification), and the prostheses can be implanted using running or interrupted techniques, as preferred.

##  

Patients suffering from mitral valve disease are at increased risk of developing supraventricular tachycardias like atrial fibrillation. Abolishment of mitral regurgitation often proves insufficient to treat this problem, and guidelines advocate concomitant treatment of atrial fibrillation in left-sided cardiosurgical procedures [8]. Indeed, development of atrial fibrillation is indicative of progressed structural atrial remodelling that can only be treated with substrate modification, using surgical ablation strategies. The most frequently used ablation modes are radiofrequency ablation (using a bipolar clamp) and cryoablation. The latter is an endocardial-only approach and can be used effectively in minimally invasive mitral valve surgery.

The malleable cryoprobe can be introduced through the working port by the table surgeon, and application of the probe to the atrial endocardium can be facilitated with the use of robotic instruments. Pulmonary vein isolation only, but also creation of a posterior left atrial wall box lesion and even a full left-sided or bi-atrial maze procedure, can be performed safely and effectively using the robotic approach, either as a stand-alone procedure or concomitant with mitral valve surgery [10]. Additionally, left atrial occlusion can be easily performed using a continuous suture.

Tricuspid valve repair is also feasible in patients undergoing robotic mitral valve surgery. When a right atriotomy is needed, double venous cannulation and temporary occlusion of the superior and inferior caval veins are needed. Drainage of the superior vena cava is established by a percutaneously introduced cannula via the right internal jugular vein. The introduction of the cannula is monitored by transoesophageal echocardiography, and the tip of the cannula is placed just under the cavoatrial junction. The same cannulation strategy can also be used when other concomitant procedures requiring a right atriotomy approach (e.g. closure of a patent foramen ovale) are needed.

##  

Related to differences in cannulation strategy and a less invasive approach with smaller surgical wounds, some potential complications related to the described techniques are possible. Awareness and quick action are essential to prevent serious complications.

Cannulation of the femoral artery can be complicated by bleeding or dissection. When the problem cannot be safely and promptly resolved, discontinuation of the procedure should be considered. Especially in larger patients, venous drainage via a single groin cannula can be insufficient to provide adequate drainage of the heart. In such cases, additional drainage of the superior caval vein can resolve the issue.

When the pericardium is opened and pericardial traction sutures are placed, particular attention is needed to prevent phrenic nerve injury. Minimally invasive mitral valve surgery is normally related to longer cardiopulmonary times compared to surgery through a median sternotomy. Adequate cardioprotection is crucial, and specific differences related to the procedure, including the fact that rewarming of the heart occurs faster in a closed chest, need to be taken into account. When a longer CPB time is anticipated, distal limb perfusion can be established with the use of a distal limb perfusion catheter.

In the case of surgical bleeding that cannot be resolved promptly, an urgent sternotomy should be performed. Special care is needed to avoid bleeding at certain crucial points during the operation, in particular during cross-clamping of the aorta, when a transthoracic clamp is used. Particular care is needed to avoid injuring the pulmonary artery of the left atrial appendage. At the end of the procedure, careful de-airing of the heart is essential to prevent neurological complications.

##  

Robotic mitral valve surgery has enriched the scope of minimally invasive cardiac surgery with the goal of minimizing surgical trauma and enhancing early patient recovery while further evolving the technical skills obtainable in the minimally invasive setting. Rather than seeing robotic surgery as a competitor to other operative techniques, the surgical society could embrace it as an important addition to the toolbox of available surgical techniques that will enable optimal patient-tailored treatment of mitral valve disease in the future. In the coming years, further development and implementation of robotic surgery is anticipated, further establishing it as a valuable treatment alternative in selected patients. New robotic systems are currently in early clinical development and will probably enrich the technological spectrum. Growing experience, establishment of appropriate teaching and proctoring programs, and collection and analysis of high-quality data are all needed to facilitate the introduction of dedicated surgical teams with appropriate quality monitoring. Robotic mitral surgery is set to offer new benefits to patients in what is likely to become a widely utilized standard treatment in the future.
